# Intrauterine growth restriction in piglets modulates postnatal immune function and hepatic transcriptional responses independently of energy intake

**DOI:** 10.3389/fphys.2023.1254958

**Published:** 2023-10-16

**Authors:** C. Amdi, C. Larsen, K. M. R. Jensen, E. Ø. Tange, H. Sato, A. R. Williams

**Affiliations:** Department of Veterinary and Animal Sciences, Faculty of Health and Medical Sciences, University of Copenhagen, Copenhagen, Denmark

**Keywords:** energy supplement, fetal development, liver metabolism, LPS challenge, intrauterine growth restriction, physiology

## Abstract

**Introduction:** Insufficient prenatal nutrition can affect fetal development and lead to intrauterine growth restriction (IUGR). The aim of this study was to investigate hepatic transcriptional responses and innate immune function in piglets suffering from IUGR compared to normal-sized piglets at 3 days of age and explore whether the provision of an energy-rich supplement at birth could modulate these parameters.

**Methods:** A total of 68 piglets were included in the study. Peripheral blood mononuclear cells were harvested for LPS stimulation, and organs were harvested post-mortem to quantify relative weights. Liver tissue was utilized for RNA sequencing coupled with gene-set enrichment analysis.

**Results:** IUGR resulted in increased expression of genes such as *PDK4* and substantial alterations in transcriptional pathways related to metabolic activity (e.g., citric acid and Krebs cycles), but these changes were equivalent in piglets given an energy-rich supplement or not. Transcriptomic analysis and serum biochemistry suggested altered glucose metabolism and a shift toward oxidation of fatty acids. IUGR piglets also exhibited suppression of genes related to innate immune function (e.g., *CXCL12*) and pathways related to cell proliferation (e.g., WNT and PDGF signaling). Moreover, they produced less IL-1β in response to LPS stimulation and had lower levels of blood eosinophils than normal-sized piglets.

**Discussion:** Taken together, our results indicate that IUGR results in early-life alterations in metabolism and immunity that may not be easily restored by the provision of exogenous energy supplementation.

## Introduction

Intrauterine growth restriction (IUGR) is defined as inadequate fetal growth *in utero*, which may predispose newborns to a variety of developmental diseases (Barker, 1998). IUGR occurs when a compromised supply of nutrients and oxygen is delivered to the fetus (Bauer et al., 2003), and this can be due to reasons such as maternal malnutrition (Salam et al., 2013), gestational hypoxia (Regnault et al., 2007), or placental inefficiency (Benton et al., 2016). This is also an increasingly pertinent issue in pig production. In countries with highly developed pig production systems, such as Denmark, a long-term trend for selective breeding for large litters has resulted in an increased number of piglets born underweight and smaller than their normal-sized littermates (Engelsmann et al., 2019; Riddersholm et al., 2021). Some, if not all, of these small piglets have suffered from IUGR during gestation due to undernutrition (Foxcroft et al., 2006; Riddersholm et al., 2021). IUGR pigs experience asymmetrical organ growth at the fetal stage, where nutrients are prioritized for organs such as the brain, heart, and liver, making them relatively larger than those in normal-sized piglets (Lynegaard et al., 2019). In addition to the difference in size, IUGR may result in altered metabolic capacity due to the altered activity of enzymes involved in nutrient metabolism (Liu et al., 2013). This abnormal metabolism especially affects the glycolysis pathway, inducing a shift to the utilization of fatty acids as an energy source (Ferenc et al., 2018).

The requirement for a newborn piglet is 200 g of colostrum as a minimum (Quesnel, Farmer, and Devillers, 2012), but IUGR piglets only ingest half of the amount, ∼100 g of colostrum (Amdi et al., 2013). The reduced intake of colostrum, in combination with an abnormal metabolism, a decreased intake of antibodies from the sow, and reduced nutrition available for the development of immune function, leaves the piglet more vulnerable to infection and sepsis (Wu et al., 2006). Previous studies on babies have demonstrated how IUGR decreases the number of leukocytes and lowers concentrations of both pro-inflammatory and anti-inflammatory cytokines when challenged with lipopolysaccharide (LPS) (Wirbelauer et al., 2010; Tröger et al., 2013), potentially compromising immunity.

Supplements with a certain nutrient composition, for example, increased glutamine content, can enhance the immune function of IUGR piglets (Zhong et al., 2012), suggesting that increased energy intake can potentially restore impaired immune function. However, most studies on the effect of IUGR on immune function and energy metabolism have focused on later postnatal time points (7–24 post gestation) (Wu et al., 2006; Zhong et al., 2012; Hu et al., 2015; Bæk et al., 2019; Amdi et al., 2020), and the effect of early-life intervention with energy-rich supplements in IUGR pigs (<3 days post gestation) is not clear.

The aim of this study was to determine potential differences in the metabolism and innate immune function between IUGR and normal-sized piglets at an early stage of life (<3 days post gestation). We hypothesized that there would be impaired immune function in IUGR piglets and that an energy supplement would have an immunostimulatory effect, resulting in a stronger immune response compared to piglets with no supplement. Moreover, we proposed that liver metabolism would differ between IUGR and normal-sized piglets, but this could also potentially be altered by providing an energy-rich supplement at birth.

## Materials and methods

The experiment was approved by the Danish Animal Experiments Inspectorate and the Danish Medical Agency (J.nr. 2018-15-0201-01515).

### Study design

In total, 68 piglets were selected from Danish Landrace × Danish Yorkshire sows (parity 1–7) that were artificially inseminated with semen from DanBred Duroc boars in a Danish commercial piggery in a 2 × 2 factorial design (IUGR/normal-sized, treatment/non-treatment). The range of piglets selected was 4–12 piglets per sow, where half of the piglets presented with IUGR and half of the piglets were normal-sized. The piglets were selected on day 0 (day of birth) and ear-tagged for identification, and only piglets with wet umbilical cords were selected. In order to reduce the sow effect, at least two IUGR (one treated and one control) and two normal-sized piglets (one treated and one control) came from the same sow. Before being removed to a nurse sow, the piglets stayed with their biological mother for colostrum consumption. Piglets were categorized as IUGR piglets by visually grading them by the modified characteristics, according to Hales et al. (2013) and Chevaux et al. (2010). IUGR piglets were defined by the following criteria: 1) a steep dolphin-like forehead and if they had one or more of the following characteristics: 2) bulging eyes, 3) wrinkles around the mouth, and 4) hair growth with no direction. Piglets without any of these characteristics or the aforementioned head shape were categorized as normal-sized piglets. The selection of a nurse sow suitable for small piglets was carried out with help from the farm staff and information on the biological sows (sow number, litter size, parity, live-born, and stillborn were noted). The temperature of the piglets was recorded, and the piglets were weighed. After measurements and selection, the piglets were placed in the heated creep area. Half of the piglets in each category (IUGR or normal-sized) were allocated to either the treatment group or the non-treatment group, resulting in a total of four groups with 15 piglets in each group. The piglets were randomly selected when being sorted into the treatment groups. The treatment groups were given a commercially available energy-rich oral paste treatment (Piggy Boost, Hatting Agro, Horsens, Denmark), a colostrum supplementation containing colostrum, fatty acids, and vitamins. Two pumps (each of 2 mL to a total of approximately 4 mL) of the treatment were given to the treatment groups twice a day at 3 h intervals.

### Blood sampling

On day 3 of age (before iron injections), the piglets were collected and transported to the research facilities at the University of Copenhagen over five consecutive weeks to gain sufficient replicates. Blood samples were collected within the first 30 min after arriving at the university. Piglets were held in dorsal recumbency, and blood was collected via jugular venipuncture using a 22-gauge needle and vacutainer tubes containing heparin (BD Vacutainer, Franklin Lakes, New Jersey, United States) for LPS challenge, serum for biochemistry, and EDTA for hematology. Blood for biochemistry was then centrifuged at room temperature for 15 min at 1.20 x (CM-6MT, ELMI, Riga, Latvia), and the resulting serum was transferred to Eppendorf tubes (Sarstedt, Nümbrecht, Germany) and immediately frozen at −20°C. The samples of all 68 piglets were collected and processed over five consecutive weeks (1–2 sampling days per week). The hematology profile was analyzed on an ADVIA 2120 Hematology System (Siemens Healthcare Diagnostics, Tarrytown, NY, United States), and cells were manually assessed. For the serum biochemistry analyses, samples were assayed using an ADVIA 1800 Chemistry System (Siemens Healthcare Diagnostics, Tarrytown, NY, United States). For cytokine production, peripheral blood mononuclear cells (PBMCs) were obtained from heparinized blood samples, as previously described (Amdi et al., 2021). PBMCs were treated with 1 μg/mL LPS for 24 h, and cytokine concentrations were then measured by ELISA using commercial antibody pairs, according to the manufacturer’s instructions (R and D Systems, United Kingdom).

### Tissue sampling

The piglets were intramuscularly anesthetized with a Zoletil mix (Zoletil 50; Virbac Denmark A/S, Kolding, Denmark) that contained xylazine (Narcoxyl 20 mg/mL; MSD Animal Health, Ballerup, Denmark), ketamine (Ketaminol 100 mg/mL; MSD Animal Health, Ballerup, Denmark), and butorphanol (Torbugesic 10 mg/mL; ScanVet Animal Health A/S, Fredensborg, Denmark). To achieve deep anesthesia, the piglets were left in an undisturbed covered box with straw. After 10 min, the piglets were euthanized with an intracardial injection of 2–3 mL pentobarbital (200 mg/mL), and the weights of the organs were recorded. The following organs were removed separately and weighed on a precision scale (Radwag, Radom, Poland): the colon, small intestine (SI), stomach, adrenal glands, kidneys, liver, lungs, spleen, heart, and brain. The stomach was weighed with content and, subsequently, after emptying it.

### RNA extraction and quantitative PCR

Approximately ∼30 mg of the liver was removed after euthanasia and stored in RNAlater (Sigma-Aldrich, Schnelldorf, Germany) at −80°C. RNA was then extracted using a commercial miRNAeasy Mini Kit (QIAGEN, Hvidovre, Denmark), following the manufacturer’s guidelines. Briefly, tissue was homogenized in QIAzol Lysis Reagent using a gentleMACS^TM^ Dissociator (Miltenyi Biotec, Germany) and filtered in an RNAeasy spin column (QIAGEN^®^), including on-column DNAase treatment. Afterward, concentration and purity were measured using a NanoDrop ND-1000 spectrophotometer (NanoDrop Technologies, DE, United States). RNA quality was assessed using the 2100 Bioanalyzer system (Aligent, Glostrup, Denmark). High-quality RNA (RIN ≥7) was used for library preparation. The cDNA libraries were prepared at BGI, Copenhagen, Denmark (www.bgi.com), and sequenced (paired-end reads of 100 bp) on a BGISEQ-500 sequencing platform. Clean reads were mapped to the *Sus scrofa* genome (*ss*11.1, ensemble ID GCA_000003025.6) using Bowtie2 (v2.2.5). Differentially expressed gene analysis was conducted using Deseq2 (Love, Huber, and Anders, 2014), with an adjusted *p*-value of <0.05 set for significance. Gene-set enrichment analysis (Broad Institute, http://software.broadinstitute.org) was used for pathway analysis. For qPCR, 500 ng of RNA was used to synthesize cDNA using a QuantiTect Reverse Transcription Kit (QIAGEN). qPCR was performed using the following cycling program on an AriaMx PCR system (Agilent): 2 min at 95°C followed by 40 cycles of 15 s at 95°C and 20 s at 60°C. Data were normalized to a reference gene (*TBP*), and relative expression and fold changes were calculated using the ^ΔΔ^CT method. Primers are listed in Table 1. Raw RNA sequence data are available at GEO under the accession number GSE226052.

**TABLE 1:** Primer sequences used for qPCR analysis.

Gene name	Forward sequence (5’–3′)	Reverse sequence (5’–3′)
*TBP*	GAT​GGA​CGT​TCG​GTT​TAG​G	AGC​AGC​ACA​GTA​CGA​GCA​A
*IGF1*	CCC​AAG​GCT​CAG​AAG​GAA​GTA	GGT​AAC​TCG​TGC​AGA​GCA​AA
*CEL*	GTG​GAC​CTG​GAA​ACC​GAC​AT	GTA​GAC​GGG​CAT​CCG​AGA​AG
*PDK4*	TGC​AAT​GAG​GGC​TAC​AGT​CG	CGG​TCA​ATG​ATC​CTC​AGG​GG
*SDF2L1*	CCA​ACA​ACC​AGG​AGG​TGA​GC	CGG​TGA​CCG​AAA​GGA​ACA​CA

### Statistical analysis

The data were analyzed in SAS (GLM procedure of SAS; SAS Inst. Inc., Cary, NC), a statistical program, according to the following model:

$$Y_{ij}=µ+\alpha_{i}+{ß}_{j}+{\left(\alpha ß\right)}_{ij}+\varepsilon_{ij},$$

where *Y*_*ij*_ is the dependent variable measured (blood characteristics and organ weights), *µ* denotes the overall mean, *α*_*i*_ denotes the effect of classification (*i* = IUGR, normal-sized), *ß*_*j*_ denotes the effect of treatment (*j* = treatment, non-treatment), (*αß*)_*ij*_ is the interaction between classification × treatment, and *ε*_*ij*_ describes the random error term. The interaction between classification × treatment was only included when significant. Means were separated using the PDIFF option and presented as least square means ± SEM and considered significant when *p* < 0.05 and a tendency when *p* < 0.10.

## Results

### Intrauterine growth restriction alters transcriptional pathways in the liver

**FIGURE 1:**
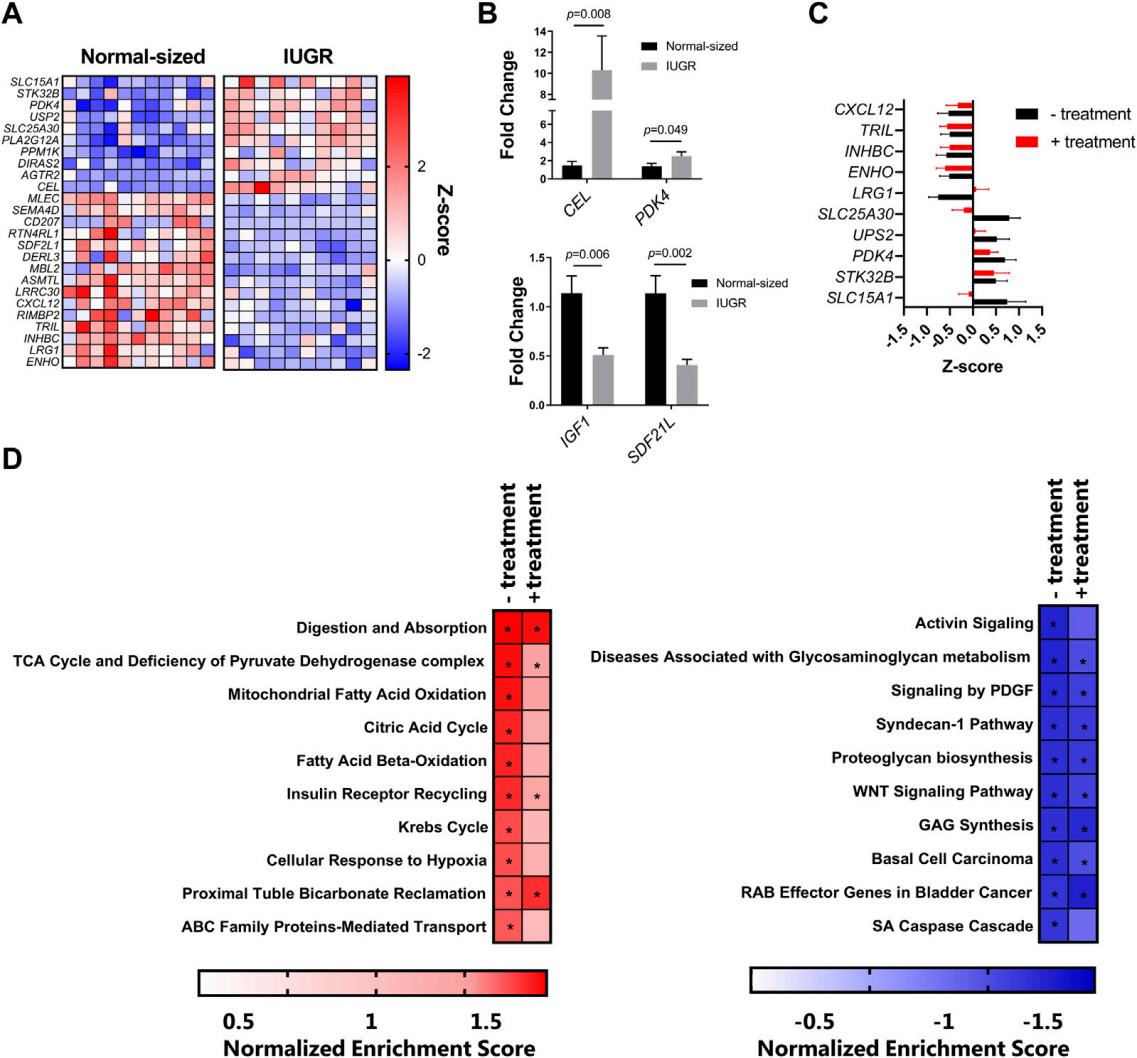
**(A)** Heatmap of the top 15 downregulated and top 10 upregulated differently expressed genes, expressed as Z-scores. The blue squares (0 to −2) are downregulated genes, and the red squares (0 to +2) are upregulated genes in IUGR when compared to the normal group. **(B)** qPCR validation of *IGF*, *PDK4*, *CEL*, and *SDF2L1* expression by qPCR. **(C)** Expression of the genes most regulated by IUGR in control piglets was mostly similar to that in IUGR piglets receiving energy supplementation (treatment). **(D)** Gene-set enrichment analysis of the top 10 downregulated and upregulated pathways (q value < 0.05) in IUGR group pigs relative to normal group pigs with or without energy supplementation (treatment).

To characterize the transcriptomic responses in the liver induced by IUGR and/or nutritional supplementation, RNA sequencing was used to identify differentially expressed genes (DEGs) and transcriptional pathways. We first questioned whether IUGR piglets had an altered liver transcriptome compared to normal-sized piglets, independent of supplementation. Deseq2 analysis identified 43 genes (12 upregulated and 31 downregulated) in IUGR pigs relative to normal controls. Figure 1A shows the top 15 downregulated and top 10 upregulated DEGs between these treatment groups (adjusted *p*-value <0.05 by DeSeq2 analysis). Notable upregulated genes included *PDK4* and *CEL*, encoding genes involved in pyruvate and lipid metabolism, respectively, suggestive of altered hepatic metabolism arising from IUGR. Downregulated genes were involved in diverse biological functions, including innate immune function (*CXCL12*), insulin signaling (*SDF2L1*), and glucose homeostasis (*ENHO*). Moreover, *IGF1* expression was also suppressed but not significantly following multiple-correction testing. For this reason, we validated *IGF1* expression, along with that of *PDK4*, *CEL*, and *SDF2L1*, by qPCR, which demonstrated significant regulation of all four genes (Figure 1B). Thus, IUGR induced significant changes in the hepatic transcriptome 3 days after birth.

In contrast, we could detect only very few genes that were regulated as a result of energy supplementation. Only two genes were significantly changed in the normal-sized treated group compared to the normal-sized control group, these being LRRC30, encoding a leucine-rich repeat protein, and HAMP, encoding hepcidin, the master regulator of iron homeostasis (data not shown). Moreover, the effect of IUGR was largely similar whether piglets received an energy supplementation or not. Expression of the genes most regulated by IUGR in control piglets was mostly similar to that in IUGR piglets receiving the energy supplementation (Figure 1C). To explore this further, we conducted gene-set enrichment analysis to detect transcriptional pathways altered by IUGR in the presence or absence of energy supplementation. In the absence of energy supplementation, IUGR resulted in a significant upregulation of pathways involved in digestion, beta-oxidation of fatty acids, the Krebs cycle, and glucose metabolism, relative to normal control piglets (Figure 1D). These same pathways also tended to be enriched in IUGR piglets receiving the supplement (relative to untreated controls), albeit at a lower level of significance, perhaps suggesting that the supplementation could attenuate some of the metabolic effects of IUGR. However, gene pathways that were suppressed were highly similar in IUGR piglets, regardless of treatment, indicating that the supplement was unable to restore impaired transcriptional responses resulting from growth restriction. Thus, IUGR resulted in substantial suppression of pathways related to cellular growth and extracellular matrix function (e.g., WNT signaling and proteoglycan synthesis), independent of energy intake, suggesting a profound effect on piglet metabolism. Collectively, these data suggest that IUGR resulted in a significant restructuring of the hepatic transcriptome with changes in energy metabolism and tissue growth and that acute energy intake has little capacity to restore these altered pathways.

### IUGR, but not energy supplementation, alters immune function

We next explored if the expression of innate immune function differed as a result of IUGR or treatment. To this end, PBMCs were isolated from all piglets and treated with LPS to assess the production of innate immune cytokines. No differences were found due to treatment (supplement/no supplement) for any cytokine or between IUGR and normal-sized piglets for IL-6 and TNFα. However, the expression of IL-1β was significantly higher in the normal-sized piglets compared to that in IUGR piglets (6261.07 vs. 4310.59; SEM 1042.23; *p* < 0.033). Thus, consistent with the transcriptomic data hinting at the suppression of some immune-related genes in the liver, peripheral immune function also appeared to be impaired as a result of IUGR.

### Blood biochemistry and hematology

Blood plasma was analyzed for albumin, total protein, basic phosphatase, alanine aminotransferase, cholesterol, creatinine, creatinine kinase, iron, inorganic phosphate, aspartate aminotransferase, blood urea nitrogen, gamma-glutamyl transferase, calcium, magnesium, sodium, potassium, glucose, and triglycerides, and no interaction between treatment and classification was found (Table 2). No differences were found in treatment; however, some effects were found for classification, which were that IUGR piglets had higher levels (48.9 vs. 40.8; SEM 1.76; *p* < 0.01) of alanine aminotransferase and blood urea nitrogen (5.2 vs. 3.1; SEM 0.75; *p* < 0.049) and lower levels of glucose (4.8 vs. 6.1; SEM 0.23; *p* < 0.001) compared to normal-sized piglets (Table 3).

**TABLE 2:** Blood biochemistry profile of IUGR and normal-sized piglets at day 3 of age.

Item	Classification		
	Normal-sized	IUGR	*p*-value
*n*	28	27	
Albumin g/L	16.0 ± 0.44	16.3 ± 0.81	0.601
Total protein, g/L	51.12 ± 1.90	50.03 ± 1.68	0.677
Basic phosphatase, U/L	3027.6 ± 156.0	3005.2 ± 210.0	0.987
Alanine aminotransferase U/L	40.75 ± 1.49	49.04 ± 1.94	0.002
Cholesterol, mmol/L	3.26 ± 0.11	3.67 ± 0.26	0.131
Creatinine, umol/L	40.79 ± 1.20	40.67 ± 1.73	0.986
Iron umol/L	4.88 ± 0.74	7.37 ± 1.62	0.166
Inorganic phosphate, mmol/L	2.07 ± 0.07	1.91 ± 0.09	0.202
Aspartate aminotransferase U/L	35.88 ± 1.43	38.76 ± 2.33	0.194
Blood urea nitrogen, mmol/L	3.07 ± 0.34	5.12 ± 1.00	0.049
Gamma-glutamyl transferase, U/L	38.36 ± 2.85	34.22 ± 2.92	0.361
Calcium, mmol/L	2.92 ± 0.05	3.00 ± 0.06	0.306
Magnesium, mmol/L	1.07 ± 0.02	1.06 ± 0.02	0.897
Sodium, mmol/L	144.46 ± 1.31	142.09 ± 1.20	0.173
Potassium mmol/L	4.00 ± 0.14	3.77 ± 0.15	0.299
Glucose, mmol/L	6.05 ± 0.24	4.76 ± 0.19	0.001
Triglyceride, mmol/L	2.47 ± 0.34	2.72 ± 0.33	0.579
Creatine kinase U/L	134.93 ± 15.05	143.74 ± 24.09	0.706

**TABLE 3:** Blood profile of IUGR and normal-sized piglets at day 3 of age.

Item	Classification		
	Normal-sized	IUGR	*p*-value
*n*	33	26	
Total leukocytes, mia/L	7.9 ± 0.39	7.7 ± 0.43	0.679
Band-shaped neutrophils, mia/L	0.25 ± 0.07	0.38 ± 0.11	0.281
Neutrophils, mia/L	5.60 ± 0.36	5.46 ± 0.35	0.789
Lymphocytes, mia/L	1.9 ± 0.11	1.6 ± 0.14	0.160
Monocytes, mia/L	0.32 ± 0.04	0.28 ± 0.04	0.483
Eosinophils, mia/L	0.06 ± 0.01	0.02 ± 0.01	0.013
Basophils, mia/L	0.02 ± 0.01	0.02 ± 0.01	0.889
Total erythrocytes, bill/L	4.09 ± 0.10	4.04 ± 0.14	0.763
Hemoglobin (HGB), mmol/L	4.93 ± 0.11	4.96 ± 0.16	0.857
Hematocrit (HCT), L/L	0.26 ± 0.01	0.25 ± 0.01	0.615
Thrombocytes, mia/L	345.0 ± 17.94	355.2 ± 18.82	0.699
MCV, fL	63.23 ± 0.65	62.88 ± 0.56	0.692
MCHC, mmol/L	19.12 ± 0.12	19.65 ± 0.47	0.231
Reticulocytes, pct. (estim.)	10.84 ± 0.64	10.20 ± 0.58	0.469
Absolute reticulocyte, mia/L (estim.)	432.4 ± 22.48	405.1 ± 23.15	0.407
nRBC	13.93 ± 1.73^a^	10.88 ± 2.03	0.255

^a^ *n* = 30, for normal; MCV, mean cell volume.

MCHC, mean corpuscular hemoglobin concentration; nRBC, nucleated red blood cell/100 WBC.

For blood hematology, there were no significant differences between treatments, and only the level of eosinophils differed between IUGR and normal-sized piglets, with IUGR piglets having lower levels than normal-sized piglets (*p* < 0.013, Table 3).

### Organ weights and growth

Table 4 presents the birth weights and body weights at day 3 for both normal-sized and IUGR piglets (there was no effect of treatment). Normal-sized piglets had an average birth weight of 1.37 ± 0.03 kg, and IUGR piglets had an average birth weight of 0.77 ± 0.02 kg (Table 4; *p* < 0.001). Normal-sized piglets had an average body weight at day 3 of 1.78 ± 0.05 kg, and IUGR piglets had an average body weight at day 3 of 0.96 ± 0.03 kg (Table 4; *p* < 0.001). There was a difference in temperature between the groups (*p* < 0.013), with the IUGR piglets having lower temperatures than the normal-sized piglets. Furthermore, there were differences between the groups in some of the organ weights, which included the following: the small intestine (SI) (*p* < 0.001), full stomach (*p* < 0.006), empty stomach (*p* < 0.003), and kidneys (*p* < 0.002), with IUGR piglets having lower organ weights than the normal-sized piglets. No differences were detected in organs such as the brain, heart, liver, or lungs. There was a significant difference in the relative brain:body ratio (rBrain), with IUGR piglets having an average higher rBrain ratio of 3.49 ± 0.11 than normal-sized piglets 2.05 ± 0.06 (*p* < 0.001).

**TABLE 4:** Characteristics of IUGR and normal-sized piglets at day 3 of age.

Item	Classification		
	Normal-sized	IUGR	*p*-value
*n*	36	32	
Birth weight (day 0), kg	1.37 ± 0.03	0.77 ± 0.02	< 0.001
Temperature day 0, °C^a^	37.2 ± 0.21	36.3 ± 0.30	0.013
*n*	36	32	
Body weight at day 3, kg	1.78 ± 0.05	0.96 ± 0.03	< 0.001
Colon weight, g	26.69 ± 1.18^b^	14.52 ± 0.62	0.114
Small intestine (SI), g	70.85 ± 3.03	51.52 ± 3.35	< 0.001
Stomach (full), g	56.81 ± 3.15	33.95 ± 2.17	0.006
Stomach (empty), g	12.08 ± 0.32	7.49 ± 0.26	0.003
Adrenal glands, g	0.48 ± 0.01	0.33 ± 0.01	0.746
Kidneys, g	15.96 ± 0.39	8.77 ± 0.31	0.002
Liver, g	64.66 ± 2.07	34.59 ± 2.09	0.540
Lungs, g	33.24 ± 1.08	20.75 ± 1.40	0.804
Spleen, g	3.35 ± 0.14	1.80 ± 0.08	0.574
Heart, g	13.73 ± 0.35	7.78 ± 0.25	0.104
Brain, g	35.76 ± 0.38	32.85 ± 0.33	0.498
rBrain, pct	2.05 ± 0.06	3.49 ± 0.11	< 0.001
rLiver, pct	3.66 ± 0.11	3.54 ± 0.17	0.859
rHeart, pct	0.78 ± 0.02	0.81 ± 0.01	0.993

^a^ Temperature, rectal temperature.

^b^ *n* = 34, in the normal piglets, two of the colon weight values were erroneous and removed from the dataset during the statistical analysis.

rBrain, relative brain-to-body ratio; rLiver, relative liver-to-body ratio; rHeart, relative heart-to-body ratio.

## Discussion

In the current study, we observed that IUGR status at birth modulated several pathways in the liver. The overall trend was that IUGR piglets displayed upregulated pathways involved in absorption, digestion, and metabolism, whilst the downregulated pathways were mostly connected to cellular morphogenesis and extracellular matrix remodeling. This may reflect an altered prioritization for nutrient partitioning to essential functions and away from growth. In contrast, very few differences were observed between piglets receiving the energy supplementation, and no notable benefits were observed compared to non-supplemented piglets. There was a significant difference in rectal temperature between the groups, with a lower temperature for the IUGR piglets than the normal-sized piglets. We have previously shown that supplementing IUGR piglets with a colostrum bolus had positive effects on rectal temperature (Amdi et al., 2017), which is crucial for survival (Baxter et al., 2008). However, the extra energy-rich supplement in this study did not contribute to any significant differences, in agreement with the findings by Schmitt and colleagues, who gave newborn piglets one extra dose of a high-fat-based energy-rich supplement (Schmitt et al., 2019).

It was notable that IUGR resulted in the upregulation of numerous genes involved in nutrient metabolism, such as *CEL* and *PDK4*. Carboxyl ester lipase (CEL) is one of four major lipases secreted by the pancreas into the duodenum (Johansson et al., 2018) and is mainly expressed in the liver and pancreas (Pfützer et al., 2002), and involved in lipid absorption (Kirby et al., 2002). *PDK4* encodes pyruvate dehydrogenase lipoamide kinase isozyme 4, a key metabolic regulator contributing to a shift from glucose to fatty acids as major energy fuel (Pettersen et al., 2019), and could indicate that pyruvate oxidation in the hepatic mitochondria is lower due to higher PDK4 and LDHA expression in IUGR individuals compared to normal individuals (Pendleton et al., 2021). In addition to lowering pyruvate oxidation, these changes are assumed to push pyruvate flux toward gluconeogenesis (Pendleton et al., 2021), possibly partly as a metabolic adaptation already determined during fetal development (Chessex et al., 1984). Notably, our results are consistent with multiple studies in a sheep model of IUGR, which indicated higher expression of *PDK4* in both hepatic and skeletal tissue, as well as markedly increased lactate production (Brown et al., 2015; Pendleton et al., 2019), suggesting that pyruvate is prevented from entering the citric acid cycle and instead is converted to oxaloacetate and, subsequently, promotes gluconeogenesis. Moreover, in accordance with our pathway analysis indicating an upregulation of transcriptional activity related to beta-oxidation of fatty acids, studies in rats have indicated that IUGR increased hepatic *Prkaa2* expression, a subunit of AMP-activated protein kinase (a master regulator of mitochondrial beta-oxidation) (Shen, Zhu, and Du, 2022). Furthermore, proteomic studies in rat hepatic tissues have demonstrated a marked enrichment of pathways related to beta-oxidation (Sarli et al., 2021). Collectively, these different studies indicate a remarkably conserved response across multiple mammalian species, whereby IUGR redirects pyruvate metabolism away from the citric acid cycle and toward gluconeogenesis, as well as promoting the oxidation of fatty acids as an energy source, likely an adaption to nutrient scarcity in the intra-uterine environment. Consistent with this, we found that IUGR piglets had lower levels of blood glucose at 3 days after birth. Glucose levels are normally around the level found in normal-sized piglets at day 3 (Engelsmann et al., 2019), suggesting that IUGR piglets take longer to reach a stable blood glucose level despite having an upregulated absorption, digestion, and metabolism pathway. In addition, sodium glucose linked transporter1 (SGLT1) was expressed relatively lower in the gut tissue of IUGR piglets compared to that of normal-sized piglets (Tang and Xiong, 2022), and GLUT4, the rate-limiting step of insulin-induced glucose uptake into the muscle (Krook et al., 2000), has been found to be decreased in IUGR piglets (Shen et al., 2018). In addition to the direct effects of nutrient scarcity, placental hypoxia may also result in metabolic reprogramming away from the citric acid cycle, and cellular responses to hypoxia were also represented in our gene pathway analysis as being upregulated in IUGR piglets.

Consistent with the growth and organ weight data, suppressed genes resulting from IUGR status included growth regulators, such as *IGF1*. Ferenc and colleagues also detected differences between IUGR and normal piglets at day 7 of age related to metabolism and found a reduction of hepatocyte numbers together with significant modifications of expression of key hormones and enzymes for protein and carbohydrate metabolism in IUGR neonates. They suggest that this might cause a predisposition to insulin resistance and obesity in adult life (Ferenc et al., 2018). In corroboration with this, the main downregulated gene pathways in IUGR piglets were related to growth. For example, the level of IGF-1 expression was downregulated compared to that in the normal piglets. In agreement, Chen and colleagues also found IGF-1 to be downregulated in the liver of IUGR piglets compared to that in normal-sized piglets (Chen et al., 2011). Tang and Xiong have recently shown that being born with IUGR affects the intestinal health of suckling piglets by altering the intestinal antioxidant capacity, glucose uptake, tight junction, and immune response (Tang and Xiong, 2022). In addition, mitochondrial dysfunction has been identified as a major factor linked to the evolution of early liver disorders and later metabolic abnormalities in IUGR individuals (Zhang et al., 2017; Rashid, Bansal, and Simmons, 2018). Nutritionally impaired growth, particularly of the liver, has long-term consequences on biosynthesis, metabolism, and host defense after birth (Cianfarani et al., 2012), and these factors, combined with high mortality among IUGR piglets, affect the whole production efficiency (Quiniou, Dagorn, and Gaudré, 2002).

Stromal cell-derived factor 2 like 1 (*Sdf2l1*) is important in liver metabolism, and in a study on mice, Sasako and colleagues found that in the liver, impaired induction of Sdf2l1 resulted in sustained endoplasmic reticulum (ER) stress, leading to insulin resistance and increased triglyceride contents, even with a normal-chow diet, indicating that dysregulation of ER stress by suppression of Sdf2l1 is a causal factor of metabolic disorders (Sasako et al., 2019).

The higher levels of alanine aminotransferase (ALT) in the blood of IUGR piglets compared to normal-sized piglets were also found by Gao et al. (2020), who also found higher levels of aspartate aminotransferase (AST) in IUGR piglets compared to normal-sized piglets at all the time points measured (days 1, 7, and 28). The authors also found that total protein content, which can be used as a biomarker of the inflammatory status in the liver, was found to be significantly lower in the IUGR piglets than in the normal-sized piglets (Gao et al., 2020). The levels of total protein found in the current study (IUGR 50.04 *versus* normal; 51.12; SEM 1.85, *p* = 0.677) were similar to the levels of total protein found in IUGR piglets in the study by Gao and colleagues, suggesting that the normal-sized piglets in the current study had lower levels than what others have reported. When looking at the size of the piglets, the piglets classified as normal-sized in the study by Gao and colleagues were 1.77 kg on day 1, compared to the normal-sized piglets in the current study, weighing 1.35 kg on day 0. A higher level of ALT and AST in IUGR piglets compared to normal-sized piglets has also been found at weaning (Niu et al., 2019). The higher level of blood urea nitrogen in IUGR piglets suggests a less efficient uptake, with more amino acids being deaminated and, therefore, excreted in the urine.

Exogenous energy supplementation did not increase the responsiveness at day 3 to an LPS challenge. Mounting an immune response is costly in terms of energy expenditure, particularly for energy and protein, because of the enhanced rate of protein turnover associated with the production of immune cells, antibodies, and acute-phase proteins (Pluske, Kim, and Black, 2018), increasing energy expenditure by 10%–15% of maintenance needs (Borel et al., 1998) and protein requirements (in particular lysine) by 7%–10% (Klasing, 2007). Differences between IUGR piglets and normal-sized piglets were observed, where the expression of LPS-induced IL-1β production was significantly higher in the normal-sized piglets compared to IUGR piglets. Interestingly, this was the same pattern we found in a previous study on IUGR pigs 24 days *postpartum* (Amdi et al., 2020), where the majority of both anti- and pro-inflammatory cytokines were similar for IUGR and normal-sized pigs, with a lower IL-1β in the IUGR group as the only exception. The observation of fewer eosinophils in IUGR piglets may also be due to the down-prioritizing of immune function compared to normal-sized piglets. Thus, the innate inflammatory responses mediated by granulocytes, monocytes, and macrophages, which may be important for resistance to infection, are partially dysregulated in IUGR piglets in the first days of life.

## Conclusion

In conclusion, IUGR piglets modulate energy metabolism and downregulate growth pathways (e.g., IGF-1) and mechanisms involved in structural development relative to normal-sized piglets. Giving an energy-rich supplement at day 0 did not alleviate the negative consequences of being born with IUGR.

## Data Availability

The datasets presented in this study can be found in online repositories. The names of the repository/repositories and accession number(s) can be found at: https://www.ncbi.nlm.nih.gov/geo/query/acc.cgi?acc=GSE226052.
